# Reconsidering Categorization-Related Inferences from Multicultural and Colorblind Prejudice Reduction Interventions

**DOI:** 10.1177/01461672251328704

**Published:** 2025-04-16

**Authors:** Manuel Becker, Sarah Teige-Mocigemba, Jeffrey W. Sherman, Karl Christoph Klauer

**Affiliations:** 1 Albert-Ludwigs-Universität Freiburg, Germany; 2 Philipps-Universität Marburg, Germany; 3 University of California at Davis, USA

**Keywords:** prejudice, social categorization, multicultural, colorblind

## Abstract

A recurring social-psychological debate surrounds the role of social categorization in moderating prejudice. In the course of this debate, researchers have examined the effectiveness of prejudice reduction interventions, reducing or reinforcing social categorization (e.g., colorblind vs. multicultural interventions). Many of the conclusions drawn in this research domain have come from studies relying on operationalizations provided by Wolsko et al. In the present research, we assessed the convergent and discriminant validity of these canonical operationalizations. Using unobtrusive measures, we provide first evidence that the operationalizations may not produce the intended differential levels of social categorization. Furthermore, the operationalizations produced unintended differences in the extent to which intergroup relations are portrayed as beneficial. These findings suggest that differential effects of these operationalizations on measures of prejudice are subject to various alternative explanations to social categorization. A better understanding of how these interventions affect prejudice might help us refine them.

Police officers having to clear school board meetings discussing Critical Race Theory in several U.S.-American cities, lethal or near-lethal attacks specifically targeting Muslims or Jews in Hanau and Halle in Germany, riots in several cities and suburbs following the deadly shooting of a young descendant of immigrants by the French police: Recent news from the Western world has been heavily dominated by intergroup conflict. Because human societies have struggled so frequently with intergroup conflict, researchers have proposed and investigated many ways to reduce prejudice and, hopefully, thereby, intergroup conflict. One common type of approach has been to frame intergroup diversity and relationships in ways that may ameliorate prejudice, and these approaches often focus on influencing social categorization, the division of the social world into distinct categories (e.g., Tajfel et al., 1971). Indeed, for decades, the status of social categorization in moderating prejudice has been a major point of contention in research on intergroup bias. On the one hand, social categorization has frequently been seen as one of the major predecessors, maybe *the* predecessor of prejudice. This perspective is based on the insight that, logically, categorization is a necessary condition for group-based prejudice. In addition, social categorization has been shown to be a sufficient condition for prejudice under certain circumstances and has been linked to prejudice in a multitude of studies (e.g., Tajfel et al., 1971; Tajfel, 1982). Accordingly, many researchers have concluded that the best way to reduce prejudice is to decrease the prevalence of social categorization. For example, categorization may be reduced by promoting a focus on individuals rather than the social categories to which they belong (*decategorization*; e.g., Brewer & Miller, 1984). In the context of ethnicity, this strategy is often referred to as adopting a “color-blind” perspective. Alternatively, categorization may be reduced by inducing perceivers to view members of different groups as belonging to a larger, more inclusive category (e.g., categorizing people as European rather than as German vs. French). This is sometimes referred to as *recategorization* (e.g., Gaertner et al., 1989).

On the other hand, it has been argued that attempts to reduce social categorization may be ineffective, at best, and, at worst, may produce a number of unintended negative consequences. This draws on the longstanding doubt that categorization of members of the most socially salient and relevant social groups can be substantially reduced at all (e.g., Hewstone et al., 1991), and some more recent evidence even suggests that the causal link between categorization and prejudice has been overstated (e.g., Park & Judd, 2005). Others have argued that members of minority groups, in particular, may benefit not from diminishing the salience of their social identities, but by recognizing and valuing them (e.g., Verkuyten, 2005). Attempts to minimize social categorization may also entitle majority groups to overlook existing structural inequalities in society or their own behavior (e.g., Apfelbaum et al., 2012), further harming members of minority groups. For all of these reasons, many researchers believe that reducing prejudice is best achieved not by reducing social categorization, but by prompting people to better recognize and appreciate diversity, thus consolidating or increasing categorization strength (e.g., Richeson & Nussbaum, 2004). This approach is commonly referred to as adopting a *multicultural* perspective.

In the past 20 years, a good deal of social-psychological research has attempted to directly compare the effectiveness of *colorblind* and *multicultural* perspectives in reducing prejudice. In terms of the social-psychological research tradition detailed above, colorblind and multicultural perspectives represent approaches that strongly differ in their assumed effect on social categorization. Guimond et al. (2014) have, for instance, verbalized this tradition by stating that colorblind interventions aim at “ignoring group membership and avoiding any reference to existing social categorizations” (p. 148), whereas multicultural perspectives imply that “categorization into distinct cultural or ethnic groups should become the rule rather than the exception” (p. 150; also see the recent meta-analysis by Whitley & Webster, 2019). The ubiquity of categorization and categorization-related inferences in research on prejudice reduction has been documented by Park and Judd (2005, p. 126) stating that social-psychological research on prejudice has been dominated by work that concludes that “to reduce intergroup hostilities, it will be necessary to minimize or eliminate social category boundaries.” The authors, again, propose multiculturalism as a possible alternative.

Researchers from many disciplines have published an impressive amount of non-experimental work on these approaches, often showing that participants that tend to subscribe to a multicultural mindset self-report less prejudice (Whitley & Webster, 2019). This accumulation of evidence underlines the potential of multicultural approaches, but given the difficulty of interpreting non-experimental studies causally, social psychological research has often strived to complement such work by experimental approaches. The bulk of experimental research comparing these perspectives has relied on short but quite elaborate messages promoting either a colorblind or a multicultural perspective on intergroup relations that were developed by Wolsko et al. (2000). In the colorblind message, participants are told that there is much scientific support for the idea that intergroup conflict can be overcome if others are judged as individuals and not by their social categories. In the multicultural message, they are told that intergroup relations could be improved if people started to be aware of, understand, and value different group experiences. After reading one of the two messages, participants are then instructed to list three reasons why the respective perspective may benefit their country, and then select from a list of responses that others have ostensibly given those that they see as most similar to their own. In the original study by Wolsko et al. (2000), the effects of the perspective manipulations were then assessed by presenting White American participants with various self-report measures of prejudice against and stereotyping of Black Americans. The authors cautiously concluded from these measures that colorblind and multicultural approaches, individually or combined, may both have their place in reducing prejudice.

The study by Wolsko et al. (2000) has attracted considerable attention (for instance, Google Scholar counts 827 citations as of December 11, 2024) and the perspective manipulations developed in that manuscript are being used in many subsequent studies that explore the differential effects of colorblindness and multiculturalism (e.g., among others, Vorauer & Quesnel, 2017; Wilton et al., 2019; note that the manipulations have sometimes been lightly redacted). Most notably, in a heavily cited study, Richeson and Nussbaum (2004) used a slightly shortened version of these manipulations and showed an advantage of multicultural interventions in reducing prejudice on a more unobtrusive measure of prejudice.

## Operationalizations of Colorblind and Multicultural Perspectives

As described above, much of social-psychological research on the effectiveness of these prejudice reduction interventions has been centrally premised on the assumption that colorblind approaches reduce categorization, whereas categorization is encouraged in multicultural approaches and instrumental in instilling a favorable view of social categories and their differences. Given that the current literature has relied almost exclusively on the operationalizations provided by Wolsko et al. (2000) in comparing these approaches, it is thus crucial that they, in fact, produce different degrees of social categorization. Note that while in this research tradition, multiculturalism is seen as the perspective that is strongly different from colorblindness in its effect on categorization, social categorization might not be the sole factor that makes multiculturalism effective. For instance, Leslie et al. (2020, p. 454) have argued in their influential recent meta-analysis that multiculturalism encompasses “learning about, maintaining, or valuing” group differences, and while all these processes are related to categorization, some of them go beyond category selection and encoding. In this vein, the validity of colorblind manipulations is tied more directly to their effects on categorization than the validity of multicultural manipulations.

As already foreshadowed above and further elaborated upon in the General Discussion, at this point, it is also appropriate to acknowledge that there is a myriad of studies (mostly) from disciplines outside psychology that examine multicultural and colorblind interventions from other angles and/or with other methods. Our research has no bearing on these studies, most importantly, because the effect of colorblind or multicultural interventions on prejudice could be mediated by many factors other than social categorization. Instead, our research is explicitly limited to categorization-related inferences. Specifically, we address the as yet unanswered question of whether the operationalizations developed by Wolsko et al. (2000) primarily and differentially affect social categorization.

Whether participants in the colorblind condition show less categorization than those in the multicultural condition has not been clearly established. Even though Wolsko et al. (2000, p. 648) infer from the outcome of their measurement procedures that participants “ignore category information” when confronted with the colorblind manipulation, they acknowledged that a possible objection to this inference lies in the susceptibility of their self-report measurement procedures to demand effects. In a literature search on all the studies citing Wolsko et al. and implementing their operationalizations, we could identify only one study aside from Wolsko et al.’s that has directly assessed the operationalizations’ effects on social categorization and, like Wolsko et al. (2000), it used a measure based on self-report (Correll et al., 2008). As noted by Wolsko et al. in their original study, the interpretation of self-report measures is complicated by the potential role of experimental demand, with participants perhaps reporting category use in line with the values expressed in the two interventions.

And indeed, there are theoretical reasons to explore the effects on categorization carefully. First, though the goal of the colorblind manipulation is to reduce categorization, it explicitly draws attention to social categories multiple times (e.g., “we are experiencing a great deal of conflict among various ethnic groups”). Subsequently, it effectively asks participants to suppress them (“must look beyond skin color”). Considerable research demonstrates that attempts to suppress unwanted thoughts, including those relating to social categories may, ironically, increase their salience in memory (e.g., Macrae et al., 1994). In addition, social categorization occurs continuously throughout the day and its underlying mental processes likely have a strong automatic or habitual component (e.g., Klauer et al., 2014). The perspective manipulation has to work against this background and the claim that a short text that repeatedly draws attention to social categories and calls for their suppression can effectively reduce categorization needs empirical evidence that is strong enough to overcome the evidence-based reservations (also see Lai et al., 2016). In sum, it is very much an open question whether the colorblind manipulation effectively reduces categorization.

A second concern with the perspective manipulations being used to compare interventions that impact categorization differently is that they may not present equally favorable outlooks on intergroup relations. For example, whereas the colorblind manipulation emphasizes the costs of immigration, the multicultural manipulation emphasizes the benefits of immigration (two exemplary excerpts from the manipulations can be found in Table 1, but we invite readers to scrutinize the complete texts as reproduced in Siy (2013). Wolsko et al. (2000, p. 637) argued that there was a “similar emphasis on positivity” in the two perspective manipulations, and that, therefore, observed differences in prejudice reduction between the manipulations cannot be attributed to one of the manipulations painting a rosier picture of interethnic relations. In line with the possible differential emphasis on positivity, emphasizing the risks versus benefits and opportunities of immigration might also introduce a difference in motivational orientation. Specifically, invoking either a promotion or prevention focus (which is, in principle, orthogonal to positivity; e.g., Phills et al., 2011) might also, in itself, lead to clear disparities in prejudice-related outcomes (e.g., Trawalter & Richeson, 2006).

**Table 1.** Excerpts From the Manipulations Developed by Wolsko et al. (2000) that May be Indicative of Differential Valence and Opportunity/Risk-Orientation.

Colorblind perspective manipulation	Multicultural perspective manipulation
We are experiencing a great deal of conflict among various ethnic groups	We are in the unique position of having many different cultural groups living within our borders
We are spending a great many resources on conflict between ethnic groups	This could potentially be a great asset because different groups bring different perspectives to life, providing a richness in styles of interaction, problem-solving strategies, food, dress, music, and art

Our present approach in testing the validity of the canonical perspective manipulations follows classical recommendations for validating treatment manipulations in assessing that “(a) the treatment manipulations are related to ‘direct’ measures of the latent variables they were designed to alter and (b) the manipulations did not produce changes in measures of related but different constructs” (Perdue & Summers, 1986, p. 318). We thus tested whether the perspective manipulations induce different levels of categorization as an aspect of convergent validity and whether they paint equally favorable pictures of intergroup relations as an aspect of discriminant validity. The extent to which the manipulations pass these validity checks thereby bears on the question of how much evidence is provided by the current literature on the role of social categorization in both these interventions and, therefore, in prejudice, more broadly. A better understanding of the mechanisms by which these (and other) interventions affect prejudice is crucial not only for a better understanding of the published findings, but also for developing effective interventions to reduce bias. Current interventions may not be ideally tuned to the aspects of these perspectives that are most critical (also see the “General Discussion” section).

## Studies 1A & 1B

In the first study (Studies 1A & 1B), we directly tested whether the colorblind and multicultural manipulations induce different levels of social categorization. To reduce concerns that demand effects might influence results, we assessed the extent of social categorization with the Who Said What? Paradigm (WSW; Taylor et al., 1978) as modified by Klauer and Wegener (1998). In the WSW task, participants are presented with pictures of people from different social categories along with statements that they purportedly had made. In a second phase, participants are shown the statements from the first phase again, along with other statements that they had not previously seen. They are instructed to indicate whether the statement they are being shown was presented in the first phase of the experiment and, if so, which of the speakers made the statement.

In the standard data analysis, the number of times a participant erroneously assigns a statement to a speaker from the same social category as the speaker that had originally made the statement (within-category error) is compared to the number of times a participant erroneously assigns a statement to a speaker from a different social category (between-category error). The ratio of within-category to between-category errors is taken as an indicator of the extent to which participants had spontaneously categorized the speakers during the first phase of the task. Because this measure reflects a variety of processes beyond categorization, including memory for the statements, memory for the individual pictures, and guessing, Klauer and Wegener (1998) developed and validated a mathematical model to account for these other processes and to improve thereby the measurement of social categorization (see also Klauer et al., 2003). The WSW is the standardized task used most frequently for the (unobtrusive) assessment of social categorization, and the category memory parameter (*d*) obtained by applying the mathematical model to WSW data is, to our knowledge, current best practice in its measurement. In the present studies, we used a powerful Bayesian hierarchical version of the WSW multinomial model (see below and the Supplemental Appendix).

To examine the effects of the colorblind and multicultural perspective manipulations on social categorization, we presented participants with one of the original manipulations (translated to German) or with one of two control manipulations and subsequently presented them with the WSW task. The two control groups comprised one group in which group members’ attention was directed towards social categories and one group in which group members were asked to perceive and assess persons as individuals. In this regard, the control manipulations were similar to the multicultural and the colorblind perspective manipulation, respectively, inasmuch as one encouraged social categorization and the other one was meant to reduce social categorization, but they differed in those ideological contents (such as that intergroup differences are intrinsically valuable) were omitted. Specifically, the first control manipulation informed participants about the significance of interethnic issues and the researchers’ interest in the participants’ impression of different ethnic groups in society. In the second control manipulation, participants’ attention was drawn to the concrete behavior of the individuals in the WSW without any mention of the social categories to which the individuals belonged. Taken together, the two control conditions thereby allow us to position the perspective manipulations and the extent of social categorization that they elicit in a range of social categorization spanned by more traditional manipulations of impression formation in terms of categorization versus individuation (e.g., Brewer, 1988; Fiske & Neuberg, 1990).

The target pictures in the WSW were presented along with an equal number of positive and negative behaviors that the persons in the pictures had purportedly performed. Subsequently, in the testing phase, participants were asked to remember which behavior had been performed by which target. All manipulations are described in depth in the “Methods” section.

In line with the reasoning outlined above, we tested whether categorization strength is reduced in the colorblind condition, compared to the multicultural condition and the control condition in which group names were merely mentioned. As an ancillary research question, we also examined whether categorization strength is reduced in the control group in which attention was directed to the actual behavior of the individuals, relative to the other three groups that mentioned social groups in different ways. Given the strength of the theoretical and empirical evidence for paradoxical effects of thought suppression, our analyses relied on directed tests.

### Methods

#### Participants

For all studies reported in this manuscript, sample sizes were determined before any data analysis, and we report all measures, manipulations, and exclusions (and methods, data and analysis files can be found in the online repository at https://osf.io/72nk4). The present studies were not preregistered, and the Bayesian hierarchical version of the WSW model implemented in Study 1 does not easily lend itself to a conventional frequentist power analysis. In previous research (Klauer et al., 2014), it was found that a group size of 40 yielded clear results in a similar analysis, so we aimed for that number for a total of *N* = 160 participants in each of Studies 1A & 1B. To be eligible for the study, participants had to be between 18 and 50 years of age, and they and both of their parents had to be German native speakers. Following our standard laboratory protocol, we agreed beforehand that participants would be excluded if: (a) they had participated in the study even though they failed to meet at least one of the inclusion criteria (*n* = 2); (b) they did not complete the perspective manipulations or indicated that they had not engaged with them or carefully read the instructions (*n* = 4); or (c) they had shown an unusual number of errors in the Evaluative Decision Task, one of two evaluative measures employed, indicating that they did not take the tasks seriously (errors in more than 25% of trials, *n* = 8; see section “Procedure”). Furthermore, the Bayesian hierarchical multinomial model we applied enables the identification of data that cannot be well represented by the WSW model, which can also be a result of sloppy engagement with the task (exclusion of *n* = 4, see below). Finally, two participants quit in the middle of the experiment. A remaining total of 318 participants took part at a laboratory in a Psychology Department at a German University.^1^

In Study 1A, data from 161 participants were analyzed; 63% of these self-identified as female, with one participant not indicating their gender. The average age was 23.3 years; 92% of participants were students (25% majoring in psychology). In Study 1B, data from 157 participants were collected; 57% of these self-identified as female, with 2 not indicating their gender. The average age was 23.6 years; 91% of participants were students (11% majoring in psychology). For each study part, a similar percentage of participants indicated that they had Turkish friends or relatives (20/17%). Table 2 presents condition-wise participant and exclusion numbers per Study part.

**Table 2.** Participant Numbers and Exclusion Numbers (In Parentheses) for Studies 1A and 1B.

Study part	Multicultural perspective manipulation	Colorblind perspective manipulation	Control group “existence of different groups”	Control group “individual behavior”
1A	41 (1)	41 (2)	39 (1)	40 (0)
1B	40 (2)	39 (4)	40 (6)	38 (4)

#### Procedure

Upon arriving at the laboratory, participants were presented with one of the four perspective manipulations described below. In Study 1A, participants were given paper-pencil versions of the manipulations; in Study 1B, they were given computerized versions. Upon completion, participants underwent the WSW task, consisting of an impression formation phase and a recognition phase. Note that in Study 1A, participants were further administered an evaluative semantic differential measure, a short self-report measure of social evaluations, after the WSW recognition phase. They were then presented with three questions about how strongly they identified with either of the two groups (Germans vs. Turks) juxtaposed in the WSW task. In Study 1B, an Evaluative Decision Task (Fazio et al., 1995), an indirect measurement task requiring quick evaluative responses to target words after a short presentation of pictures of German and Turkish men, was completed in between the impression formation and recognition phases of the WSW task. The evaluation measures were collected for the purpose of another line of research (Becker et al., 2025). As such, we will only briefly sketch the results of the evaluation measures in the Supplemental Appendix. Raw data from all measures can be found on the online repository. The studies ended with participants entering their demographic data, including the above-mentioned question about whether they had any Turkish friends or relatives. In Study 1A, all participants were compensated with 3.50 Euro or partial course credit. In Study 1B, participants were compensated according to their performance in the WSW recognition phase, averaging about 6 Euro (see the Supplemental Appendix).

#### Tasks and Materials

Following the original manipulations used by Wolsko et al. (2000, Studies 2 & 3) and Richeson and Nussbaum (2004), manipulations consisted of three parts: Participants were first presented with a text proposing that a majority of researchers either endorse the colorblind or multicultural perspective, and then detailing how the reader can use this knowledge to improve intergroup relations. Participants were then asked to list three reasons why their respective perspective may benefit their country. Finally, they were asked to select from a list of responses that other people have ostensibly given to the last task those responses that they see as most similar to their own. This list of responses included arguments that were further strengthening the respective perspectives participants had been presented with. We closely translated-backtranslated the slightly shortened manipulations from Richeson and Nussbaum (2004) and cautiously adapted them to the German context, where we deemed it absolutely necessary.^2^

As described above, we developed two control group manipulations with very similar task demands. In the first control group, participants were presented with a text proposing that a majority of researchers think that the relations between people from different ethnic backgrounds are a very important topic. Participants were then asked to list three contexts in which people from different ethnic backgrounds might meet in Germany. Finally, they were asked to select from a list of responses that other people have ostensibly given to the last task those responses that they see as most similar to their own. Like for the control group implemented by Wolsko et al. (2000; Richeson & Nussbaum, 2004, did not implement a control group), the control condition draws attention to the ethnicity dimension; it also keeps structure and task demands similar to the conditions with perspective manipulations.

This was also true for the second control group. Here, participants were presented with a text proposing that a majority of researchers think that one can draw profound inferences about people from the actual behaviors that they show. Participants were then asked to list three behaviors they have observed in other people before. Finally, they were asked to select from a list of responses that other people have ostensibly given to the last task those responses that they see as similar to their own.

##### “Who Said What?” Task

As target stimuli in the WSW, we used twelve portraits of males selected from a stimulus pool provided by Singmann et al. (2013) that had previously been validated as indicative of German or Turkish ethnicity and were matched for emotional expression and attractiveness (see the Supplemental Appendix, also see Teige-Mocigemba et al., 2017). We decided to use Turks as the outgroup category not only because they are Germany’s largest ethnic minority, but also because there is rather clear evidence for prejudice against Turks for German participants in general and for our participant pool in particular (Teige-Mocigemba et al., 2017).

The behaviors were positive and negative versions of 96 behaviors validated by Ehrenberg et al. (2001). To illustrate, a positive version of one behavior reads “almost never jokes at the expense of others,” and the negative version reads “often jokes at the expense of others.” Pilot testing indicated that both the positive and negative behaviors were equally stereotypic of Germans and Turks, see the Supplemental Appendix.

The positive and negative behaviors were randomly divided into two sets of 48 behaviors, respectively, with the first half presented with the portrait stimuli in the impression phase and the second half added as distractors in the recognition phase. Accordingly, four behaviors were assigned to each of the six pictures of German men and each of the six pictures of Turkish men in the impression formation phase. The pictures (360 × 480 px) were first presented for 1.5 s in the center of the screen, followed by the presentation of the behavior along with the picture for 8 s. Participants were told to follow the presentation of the behaviors and form an impression of the people that were presented. The valence of the behaviors was counterbalanced so that each portrait was presented with two positive and two negative behaviors. The pictures were presented in turn, so that in a first round, each of the twelve pictures was shown with one behavior, followed by the second round, and so on. The order of pictures was determined randomly in each round anew.

Upon completion of the first phase, participants were informed of a surprise recognition task. In random order, participants were presented with the behaviors from the impression formation set and the distractor set. They were instructed to indicate whether they had encountered the specific behavior in the impression formation phase. If they responded that the behavior had been previously shown by clicking on a button labeled “old,” they were presented with a screen containing smaller versions of the twelve pictures in two rows of six pictures and asked to select the picture that was previously presented as having shown the specific behavior. After choosing one of the pictures, the next behavior was shown. If participants responded that a behavior had not been previously shown by clicking on a button labeled “new,” the next behavior immediately followed.

#### Analysis

The main analysis was conducted by means of a hierarchical Bayesian multinomial model analysis of the WSW measurement outcome that treats participants and stimuli as random factors (for a comprehensive description of the model, see Klauer & Wegener, 1998; and Klauer et al., 2014; for the underlying statistical model, consult Klauer, 2010; and Matzke et al., 2015). The analysis outcome consists of individual parameter values assessing the likelihood of different assumed latent mental processes to occur. In the following, we will only present some basic information needed to understand the model and our inferential logic. For further information on the model and our modeling decisions, please consult the Supplemental Appendix at the online repository.

As described above, the model allows one to assess social categorization (in the form of category memory) disentangled from other mental processes (see Table 3). In applying the model, a number of decisions have to be made, which also influence the number of parameters that are estimated in the model. As detailed in the Supplemental Appendix, a model in which person memory parameters were allowed to vary as a function of stimulus ethnicity, but all other parameters were collapsed across ethnicity, provided the best relative fit for the data. We therefore chose this model for the following analyses of the individual study parts 1A&B, and of a joint analysis of their data. After the exclusion of four participants with inadequate individual model fit statistics, model checks for the individual studies and the joint analysis were satisfactory.

**Table 3.** Parameters From the WSW Model as Applied in Studies 1A&B.

Parameter	Meaning	Estimates probability
I	Item memory	. . .that a behavior is detected to be an “old” behavior that has been shown in the impression phase or a “new” distractor
C	Person memory	. . .that the person that has shown the specific behavior is remembered for a behavior correctly detected as old
D	Category memory	. . .that the ethnicity of the person that has shown the specific behavior is remembered for a behavior correctly detected as old, for which there is no person memory
B	Item status guessing	. . .that a behavior is guessed to be old if there is no item memory for the behavior
A	Category guessing	. . .of guessing that the behavior was shown by a German instead of a Turk in guessing without memory. The probability to guess that the behavior was shown by a Turk corresponds to 1-a

*Note*. WSW = Who Said What?.

The Supplemental Appendix also contains a description of the interpretation of the outcome of the Bayesian hierarchical analysis. The hierarchical Bayesian approach yields Highest Density Intervals (HDIs) for all parameter estimates, similar in interpretation to standard confidence intervals. More importantly for the present purposes, it also allows for hypothesis tests for equality between parameters across the different experimental conditions. We present these comparisons as a *p* value. For the present purposes, the key comparison tested for equality of the category memory parameter d in the multicultural and colorblind conditions. We also compared the three conditions in which, in our reading, attention was directed towards social categories with the condition in which attention was directed towards individual behavior.

### Results

In light of the Bayesian nature of our analyses, and to give a succinct and informative overview of our studies, we present our analyses jointly, although we also report separate posterior values for the individual studies. A complete presentation of the analyses of the studies follows the technical details of the model in the Supplemental Appendix. Importantly, as described above, model fit was acceptable for both models and, overall, behavior, person, and category memory parameters were similar in both experiments.

**Figure 1.**
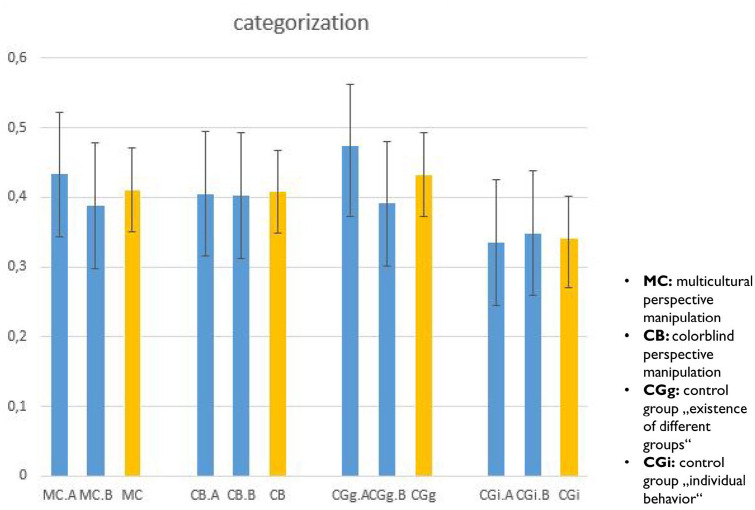
Effects of perspective manipulations on category memory. *Note.* Category memory estimates for Study 1A (left bar), Study 1B (middle bar), joint analysis (right bar) per condition (in order, MC = multicultural, CB = colorblind, CG_g_ = control group_group_, CG_i_ = control group_individual_). Error bars denote 95% HDIs.

Figure 1 shows the category memory estimates, *d*, along with 95% HDIs, as a function of the participants’ perspective manipulation condition. As can be seen, categorization is descriptively highest in the control condition similar to the one used by Wolsko et al. (2000), in which participants were merely reminded of the existence of different groups in society. This categorization estimate is, however, very similar to the conditions in which participants were presented with the multicultural and colorblind perspective manipulations. The control group in which participants were told about the diagnosticity of individual behaviors (control group_individual_) showed the smallest amount of categorization. As foreshadowed by the very similar parameter estimates, there was no evidence for decreased categorization in the colorblind compared to the multicultural condition in a posterior test, *p* = .467 (Study 1A: *p* = .322; Study 1B: *p* = .603). In contrast, there was evidence in a posterior test for decreased categorization in the control group_individual_ in comparison to the other three conditions, *p* = .018 (Study 1A: *p* = .024; Study 1B; *p* = .186).

To assess whether our data provided evidence for the invariance between the effects of the multiculturalism and colorblind manipulation conditions on categorization strength, we computed a Bayes factor. To allow for a conservative estimate, we implemented a prior implying a weak effect. This analysis provided moderate evidence for the null hypothesis, *BF*_01_ = 4.7 (cf., e.g., Wetzels & Wagenmakers, 2012). In contrast, a repetition of this analysis for the contrast between the control group_individual_ and the other three conditions provided moderate evidence for a decrease in categorization, *BF*_10_ = 3.4.

### Discussion

Study 1 indicates that the perspective manipulations do not induce different levels of categorization. Over both studies, we could not confirm that the commonly used color-blind perspective manipulation produces lower levels of categorization than the multicultural perspective manipulation. Instead, a structurally similar, newly created control group in which participants were told about the diagnosticity of individual behaviors showed decreased categorization estimates. When analyzed individually, however, this decrease was not credibly different from zero in the Bayesian posterior test in Study 1B. This could be indicative of the fact that the effects from these manipulations on the course of mental processes are likely rather small (also see “General Discussion” section).

One limitation of Study 1 is the reliance on only one social categorization measure (WSW). As foreshadowed above, however, the outcome of the WSW is the current best practice in the measurement of social categorization and we are not aware of any other measurement procedures that have also been as thoroughly validated, especially with regard to their susceptibility to demand effects. Once such measures become available, it would certainly be worthwhile to reevaluate the present findings.

Another possible objection to these findings could be that in Study 1B, the Evaluative Decision Task in between the impression formation and the recognition phase may have influenced the categorization measurement. We do not think that this objection is especially plausible, as memory performance was very similar across the two studies, see the Supplemental Appendix.

## Study 2A

In Study 1, we tested whether the perspective manipulations differ in the levels of categorization that they induce for a check of convergent validity of the manipulations. In Study 2A, we addressed an issue of discriminant validity, namely that the manipulations do not differ in how favorably they portray different ethnic groups. If they do differ, this would complicate categorization-related interpretations of differences between the colorblind and multicultural perspective manipulations.

Because this issue concerns the participants’ impressions, we simply presented participants with either of the two core text vignettes of the perspective manipulations and asked them to rate how positively or negatively the vignette describes people with different ethnic backgrounds living together, and how clearly it presents the opportunities and risks of people with different ethnicities living together.

### Methods

#### Participants

We recruited the same number of participants per condition as in Studies 1A and 1B, applying the same inclusion and exclusion criteria, where applicable—with the exception that we relaxed the age criterion such that only participants below the age of 18 years were excluded from the Study (since there were no cognitively taxing memory tasks in this study). In Study 2, participant exclusions were all due to subjects participating in the study despite failing to meet at least one of the inclusion criteria (*n* = 4 in the multicultural, *n* = 3 in the colorblind condition). Data from 80 participants remained, 40 per group in a between-subjects design; 75% of these self-identified as female. The average age was 23.4 years; 86% of participants were students (and 5% self-offering that they were Psychology students). A sensitivity power analysis for the two analyses presented below (two-tailed *t*-tests for independent means, α = .05, β = .80; computed with G-Power 3.1, Faul et al., 2007) indicated that the minimum effect size that could be detected was *d* = 0.63, which seems reasonable, given that the differences between the two manipulations seem rather pronounced. Participation was compensated with sweets.

#### Materials and Procedure

The study was conducted as a paper-pencil study. Participants were handed a double-sided sheet of paper, the original German version of which is available at the online repository. On one side of the paper, the core text of one of the two perspective manipulations was printed, the respective manipulation being randomly determined. Above the text was a short instruction text asking participants to carefully read the instruction and then answer some questions on the backside. The (English translation of the) instruction on the next page read:

The present text has been implemented in several scientific studies to encourage a certain perspective on different social groups living together. We are interested in your evaluation of the text with regard to the following questions. Please attend only to the specifics of the present text—this study is not about whether you share the underlying values/ ideas presented in the text.

Participants were then asked to rate on a 7-point rating scale ranging from “*very negative*” to “*very positive*” the question: “How does the text, in your opinion, portray people with different ethnic backgrounds living together”? Then, they were asked to rate on two 7-point rating scales ranging from “*not at all*” to “*very much*” the questions: “To what extent does the text illustrate the opportunities of people with different ethnic background living together”? and “To what extent does the text illustrate the risks/ possible problems of people with different ethnic background living together”? The questionnaire closed with the demographic data.

### Results

**Figure 2.**
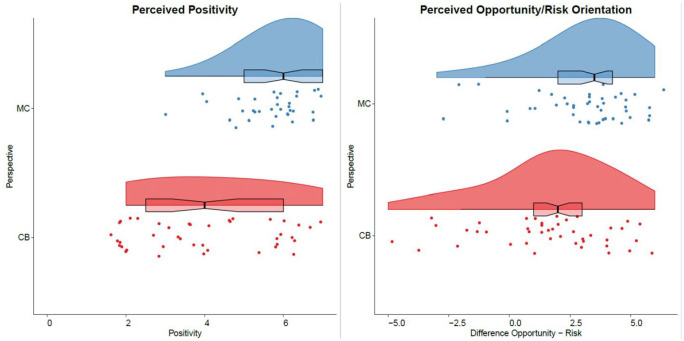
Perceived valence and opportunity/risk orientation of perspective manipulations. *Note.* Raincloud plots (via Allen et al., 2019) for judgments of positivity and opportunity-risk-orientation of the multicultural (MC; plots printed in blue) and colorblind (CB; plots printed in red) perspective manipulations.

The text from the multicultural perspective manipulation was seen as portraying people with different ethnicities living together as pronouncedly more positive (*M* = 5.95, *SD* = 0.99) than the text from the colorblind perspective manipulation (*M* = 4.05, *SD* = 1.74), *t*(78) = 6.01, *p* < .001, *d* = 1.34, CI_d_ = [0.86, 1.83]; see Figure 2. Analyses for this study were performed with IBM SPSS Statistics (Version 29).

For the analysis of the perceived opportunity/risk orientation of the manipulations, we first subtracted the perceived risk orientation from the perceived opportunity orientation. This index can be seen as an indicator of how many participants perceive the text as relatively more opportunity than risk oriented. The text from the multicultural perspective manipulation was perceived as more strongly illustrating the opportunities relative to risks of people with different ethnic backgrounds living together (*M* = 3.20, *SD* = 2.11) than the colorblind perspective manipulation (*M* = 1.53, *SD* = 2.56), *t*(78) = 3.19, *p* = .002, *d* = 0.71, CI_d_ = [0.26, 1.16].

### Discussion

Study 2A provided strong evidence that the multicultural perspective manipulation gives a rosier outlook on interethnic relations than does the colorblind manipulation. Stronger prejudice-reduction effects of the multicultural versus the colorblind manipulation (e.g., Richeson & Nussbaum, 2004) might thus reflect differences in the manipulations’ positivity or motivational orientation rather than differences in the participants’ propensity to apply category information, per se. Accordingly, the extent to which these manipulations affect prejudice via social categorization is unclear.

As described above, the differential positivity of the two instructions may be seen as a logical consequence of their different underlying principles: By its nature, multiculturalism might hinge more on a positive portrayal of the outgroup. In contrast, the colorblind perspective should draw little attention towards categories, which certainly complicates communicating positivity towards the outgroup in an equal manner.

We do not believe this is an unsurmountable problem. For instance, it is certainly possible to envision a manipulation that is much more positive and with a stronger focus on opportunities rather than risks of immigration. Consider, for example, the first row of Table 1: The sentence from the multicultural condition (which describes different groups as an opportunity for good things) could easily be used in the colorblind condition (which at present describes different groups as an opportunity for bad things) without increasing categorization relative to the original sentence. It also seems likely that highlighting the dignity and value of all human beings could be included in a more positive version of the colorblind manipulation.

Thus, we developed new versions of both the colorblind and the multicultural manipulation texts and put them to a test. Our goal in the present study was mostly a proof-of-concept: Are we, with our combined knowledge in this research domain, able to draft new manipulations that pass the valence-related manipulation checks, while staying true to the original ideologies? If that is the case, the objection that differential positivity is a necessary feature of categorization-related interventions would seem less plausible to us.

## Coda (Study 2B)

In revising the original manipulation texts, we tried to stick to the original messages as closely as possible. In addition, we tried to align the wording and structure of the manipulations wherever this did not conflict with the differences between the ideologies. More importantly, to account for our concerns spelled out above, we took care to describe the relationships between different people in a similarly positive way and emphasize opportunities (rather than risks) of people’s diversity to a similar extent. Likewise, we greatly reduced references in the colorblind condition that raise the salience of different ethnic groups, eliminated all calls for the suppression of ethnicity-related thoughts, and replaced them with a focus on valuing each other and each other’s individuality. The new versions of the manipulations and their translation can again be found in our online repository (https://osf.io/72nk4).

In Study 2B, we thus tested the two newly developed manipulations with the same dependent variables that were used for the original manipulations in Study 2A, and additionally ran the original manipulations again as a direct replication to be able to directly compare the results between the new and the original manipulation texts. Participants were also presented with an additional manipulation check, to ensure that the new manipulation texts convey the respective ideologies in a similar manner to the classical manipulation texts.

### Methods

#### Participants

We aimed to recruit the same number of participants per condition as in Study 2A, applying the same inclusion and exclusion criteria, where applicable. Participants were able to participate in a paper-pencil version of the study as in Study 2A, or in a digital version implemented on SoSciSurvey. They were recruited as an ad hoc sample in exchange for sweets, on a university recruitment platform in exchange for course credit, or on Prolific, where they were compensated with £1,50. We decided that for online recruitment, communicating the parental language criterion might further increase risks related to participant self-selection, so that exclusion of these participants was generally performed ex post instead of ex ante as in the previous studies. As a consequence, a rather large number of participants (*n* = 42) were excluded based on this criterion. In addition, *n* = 6 participants from the university recruitment platform erroneously participated more that once, in which case we only included their first, naïve run in the dataset. Due to the partial online recruitment, we slightly oversampled, such that data from 167 participants remained, with *n* = 45 participants assigned to the new “colorblind” manipulation, and *n* = 42 participants assigned to the original multicultural manipulation, and *n* = 40 participants to each of the remaining two conditions. 58% of participants self-identified as female, their average age was 26.3 years, and 70% of participants were students (and 4% self-offering that they were Psychology students).

#### Materials and Procedure

Apart from the subset of participants that were able to take part in the study online, the experimental procedures of Study 2B exactly copied the procedures of Study 2A, with one further exception^3^: As mentioned above, we implemented another short manipulation check to ensure that the new manipulation texts are similarly perceived by participants in terms of the core message of the perspective manipulation. Thus, after participants completed the three questions from Study 2A (and thus all original experimental measures that had been presented in Study 2A), they were presented with a fourth question. We asked participants to evaluate whether the manipulations were more in line with a summary of the colorblind ideology or with a summary of the multicultural ideology. The (English translation of the) instruction read: “Please classify which of the two perspectives that anchor the scale below is more strongly underlined by the text you just read.” Participants responded on a 7-point Likert scale ranging from a summary of the colorblind perspective (“The equal value of people from different ethnic groups is emphasized because each person is an individual with their own advantages and strengths”) to a summary of the multicultural perspective (“The diversity of people from different ethnic groups is emphasized because each group has its own advantages and strengths”). The questionnaire closed, again, with the demographic data.

### Results

For the positivity ratings, the opportunity-risk-orientation ratings, and the additional manipulation check, we calculated simple t-tests equivalently to Study 2A, both for the original and the newly developed versions of the manipulation texts separately. For the analyses related to the classical manipulation texts, this serves as a replication for Study 2A. For the analyses related to the newly developed manipulation texts, this assesses whether they are perceived in a way that makes them pass our manipulation check.

**Figure 3.**
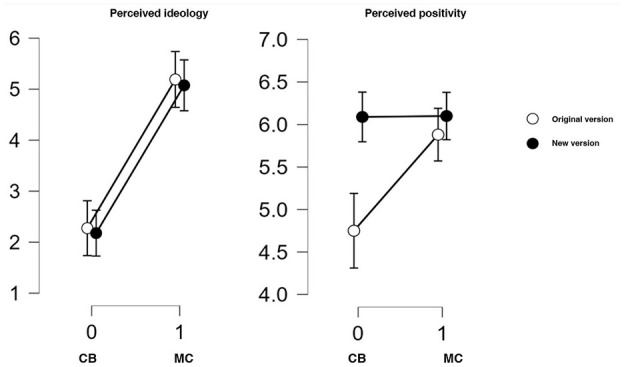
Perceived ideology and valence of perspective manipulations. *Note.* Mean judgments of perceived core message ideology and perceived positivity of the multicultural (MC) and colorblind (CB) perspective manipulations in their original and the newly-developed version, respectively.

As can be seen in Figure 3, the original version and the newly-developed version of the colorblind manipulation texts are almost identical in how similar to the summary of the colorblind ideology they are perceived (*M*_CBoriginal_ = 2.28, *SD* = 1.68; *M*_CBnew_ = 2.18, *SD* = 1.5). Likewise, the two multicultural manipulations texts are closely aligned in how similar to the summary of the multicultural ideology they are perceived (*M*_MCoriginal_ = 5.19, *SD* = 1.76; *M*_MCnew_ = 5.08, *SD* = 1.56). Accordingly, the t-tests for the difference between the colorblind and multicultural manipulation texts document a pronounced difference for both the original and the newly-developed texts (for the original manipulations, *t*[80] = 7.676, *p* < .001, *d* = 1.7, CI_d_ = [1.38, 2.41]; for the newly-developed manipulations, *t*[83] = 8.735, *p* < .001, *d* = 1.9, CI_d_ = [1.19, 2.22]).

While the original and new versions of the manipulation texts seem to be remarkably similar in how well they portray the core message of the ideologies, they differ in their portrayal of intergroup relations. As can be seen in Figure 3, we replicate the finding from Study 2A that the original multicultural perspective manipulation is portraying people with different ethnicities living together as pronouncedly more positive (*M* = 5.88, *SD* = 1) than the text from the colorblind perspective manipulation (*M* = 4.75, *SD* = 1.373), *t*(70.812) = 4.257, *p* < .001, *d* = 0.95, CI_d_ = [0.49, 1.4]. In contrast, the judgments of the valence of the two newly developed manipulations are virtually identical (*M*_MCnew_ = 6.10, *SD* = 0.87; *M*_CBnew_ = 6.09, *SD* = 0.97; *t*[83] = 0.06, *p* = .96, *d* = 0.01, CI_d_ = [−0.41, 0.44]).

Finally, the difference score for the perceived opportunity/risk orientation shows a significant difference between the ideologies for the original manipulations (*M*_MCoriginal_ = 2.55, *SD* = 1.8; *M*_CBoriginal_ = 1.6, *SD* = 2.22; *t*[80] = 2.131, *p* = .036, *d* = 0.47, CI_d_ = [0.03, 0.91]), but not for the newly-developed manipulations (*M*_MCnew_ = 2.85, *SD* = 1.93; *M*_CBnew_ = 3.13, *SD* = 2.31; *t*[83] = −0.609, *p* = .544, *d* = −0.13, CI_d_ = [−0.56, 0.29]). Thus, in contrast to the original versions, the newly developed manipulations also pass this manipulation check: There are no indications that the new multicultural text might be more pronouncedly, illustrating the opportunities relative to risks of people with different ethnic backgrounds living together.

### Discussion

At the end of the Discussion of Study 2A, we presented the possibly most impactful objection against our claim that there might be unintended effects in the positivity of how ethnic groups are portrayed in the two classical manipulations of colorblindness and multiculturalism: Namely, that a valence differential might be necessary to convey the two ideologies, especially if the colorblind manipulation draws little attention to ethnic groups. Since we did not believe this to be an insurmountable problem, we developed new manipulation texts, and assessed whether these new versions would be similarly perceived in terms of their core ideological message as the original ones, while passing our test of discriminant validity, namely that the texts do not differ in how positively they portray different ethnic groups. In the manipulation checks we implemented, a completely unambiguous picture emerged: Our newly developed manipulations were equally able to convey the core colorblind and multicultural messages as the original ones, while presenting relations among ethnic groups equally favorably.

## General Discussion

Against the background of a long-simmering debate in psychology on the role of social categorization in prejudice and deep-running societal debates on how to best combat intergroup biases and conflict, Wolsko et al. (2000) developed operationalizations of two ideological perspectives—multiculturalism and colorblindness—that differ in their assumed effects on social categorization. The impact of these operationalizations has been thoroughly examined and they have frequently been implemented to investigate the role of social categorization in prejudice. In the present work, we revisited two central theoretical assumptions of this research tradition: namely, that the manipulations induce different levels of categorization and portray intergroup relations equally positively. These two assumptions were tested in two validity checks, the first being a check of convergent validity that the intended effects on social categorization occur, and the second being a check of discriminant validity that there are no unintended effects in the positivity of how relations among ethnic groups are presented. The present experiments provided no evidence for either assumption. The conditions did not differ in extent of induced social categorization but did differ in terms of valence, with the multicultural prompt being viewed more positively.

### Convergent Validity Check

The outcomes of the two validity checks have different consequences. Consider first the finding that the manipulations did not produce differences in social categorization. This does not undermine the possibility that the colorblind and the multicultural manipulation differ in their effects on prejudice, but it does indicate that any observed differences in prejudice between the two conditions are not the result of differing levels of categorization. This result is especially problematic for the colorblind manipulation text, given that it epitomizes the ideal of tackling prejudice by reducing categorization, which it did not even achieve in comparison to a control condition in which category salience is raised. In contrast, it might be less problematic for the multicultural manipulation text, given that its category estimate is similar to the control condition in which category salience is raised. More importantly and as mentioned above, the description of its core ingredients has often added factors beyond elevated categorization, for instance, the appreciation of different groups. Hence, one could argue that the original multicultural text might be a valid operationalization of the multicultural perspective. We can nevertheless conclude based on our results that it is questionable to attribute differences in outcomes associated with the two operationalizations to differences in social categorization. Instead, such differences would have to be attributed to other factors with respect to which the two operationalizations differ. In the study revisited in the next section, we focus on one such difference, affective tonality.

It is worth noting that the model’s measure of social categorization was, in fact, sensitive to differences across conditions: The “individuation” control condition, which focused on behavior without also invoking categories, did yield lower estimates of categorization. This highlights an important distinction within prejudice-reduction approaches that aim at reducing social categorization, between those approaches that emphasize intergroup assimilation and disuse of categories, and approaches directed more toward individuation, which seek to reduce prejudice via a focus on individual features and behavior. Our attempt in revising the colorblind manipulation was in line with the latter approaches, but the results from Study 1 might encourage future researchers to tune future manipulations even further toward an individuation framing.

### Discriminant Validity Check

The second finding suggests that the contrast between the colorblind and the multicultural manipulation lacks discriminant validity. Specifically, the perspectives presented in the manipulations are confounded with how positively relations among social/ethnic groups are portrayed in the two conditions. The confound between perspectives and valence in these manipulations is problematic for any assertion that differential effects of the two perspective manipulations can be attributed to variation in social categorization. The higher favorability of the multicultural prompt also raises the possibility that demand effects could drive observations of reduced prejudice in this condition, particularly on self-report measures: Participants may simply report more favorable intergroup attitudes because they think they should.

Demand effects are problematic if we trace back the interventions’ effect on self-reported prejudice measures to their differences in categorization, but researchers have identified means to deal with them (see the Conclusion). Given social psychology’s longstanding focus on categorization, it might be fruitful to consider that while multicultural interventions have often been described in reference to their effects on categorization, this might not be the best angle to look at them. For instance, the instruction of participants to view different ethnic groups in a positive light might mainly drive the downstream effects of the multicultural prompts. In this view, consolidated categorization is at most a byproduct of multiculturalism, but interventions consolidating categorization are still preferred, because they make it easier to cast different ethnic groups in a positive light. If researchers make this reasoning explicit, they could test the effects of manipulations casting different ethnic groups in a positive light versus reducing categorization. Given that our Study 2B provides substantial evidence that versions of these texts can be developed that are much less different in terms of valence, a steelman version of this test probably involves a colorblind manipulation that does not actively promote the risks of different ethnic groups in a country. In any case, the current manipulations that do not differ in their effects on categorization, but do differ strongly in their portrayal of different ethnic groups, are not an adequate test of the potential of tackling categorization for reducing prejudice.

### Factors Contributing to the Effectiveness of Ideology Perspectives

The concern with valence differences as a key factor in observed responses to the classic multicultural and colorblind manipulations can be put into a larger context. As foreshadowed above, research on multicultural and colorblind frames is not limited to social psychological research. Researchers from other disciplines, as well as social psychologists, have speculated about other possible psychological constructs beyond social categorization operating in these ideologies (or, at least, in specific operationalizations of these ideologies; see, e.g., Rosenthal & Levy, 2010). Though this is intriguing research that may generate new approaches to prejudice reduction, it is also detached from the question at the heart of our research: Do these widely used manipulations indeed affect categorization as they are typically posited to do? If mechanisms unrelated to categorization govern the effects of these frames, research using these frames obviously does not speak to the question whether interventions successfully discouraging people from categorizing or encouraging people to categorize affect prejudice. To be absolutely clear about this important point, the present research only addresses the validity of categorization-related inferences from research implementing these frames. It very much leaves open the possibility that (a) a multicultural (or colorblind) mindset in general, (b) multicultural (or colorblind) interventions that are successful in altering categorization, and (c) even multicultural (or colorblind) interventions that are not successful in altering categorization may very well nevertheless be successful in reducing prejudice.

It has to be noted that one additional factor possibly operating in the present study that is worth explicitly discussing is our implementation of a translated version of the manipulations in Germany. If the exact operationalization of the manipulations only affects categorization in U.S.-Americans, results could be concealed in Germany. However, we are not aware of any suggestion of cultural differences, either in theory or data, in responding to frames encouraging or discouraging categorization (also see Whitley & Webster, 2019), and research using translations of Wolsko et al.’s (2000) perspective manipulations outside of the United States is regularly conducted and published (e.g., Costa-Lopes et al., 2014). Thus, we have no a priori reason to believe that these manipulations should be more or less effective in different countries. More generally and reiterating the logic from above, the disputes in this classic social-psychological line of research have always centered on the universal effects of categorization on human information processing. As such, any claim for nation-specificity needs to be made explicitly and corroborated empirically. It also is worth noting here that several recent studies that have assessed mechanisms operating in social categorization have shown similar effects in German and U.S.-American samples (e.g., Flade et al., 2019; Klauer et al., 2014).

## Conclusion

Our focus in the present work was a close examination of the common experimental inductions of multicultural and colorblind perspectives in prejudice reduction research (Wolsko et al., 2000). To the extent that these operationalizations are used to examine the impact of increased versus decreased use of social categories, the failed validity checks presented here pose significant problems. They challenge the view that effects of the operationalizations are caused by differences in social categorization. Instead, the evidence suggests that effects of the operationalizations may be caused by differences in positivity and, possibly, demand characteristics. To defend against such demand characteristics, researchers might be well-advised to either use dependent variables that are difficult to control or allow for sufficient time to pass between intervention and measurement so that demand effects have a chance to have dissipated (also cf. Lai et al., 2016).

In light of these problems, we have taken a first step in developing text-based colorblind and multicultural perspective manipulations that are similar in valence but differentially and meaningfully affect categorization. These or similar manipulations (see the individuation condition) may be tested for their effects on categorization and prejudice as a logical next step. Alternatively, researchers believing that multicultural and colorblind interventions need not (or not only) differ in social categorization, but also in terms of other psychological constructs, might make these factors explicit as a first step to develop new manipulations focused on these other constructs while equating the manipulations on factors believed to be irrelevant (for encouraging work in this direction, see, e.g., Leslie et al., 2020; Rosenthal & Levy, 2010; also note that in terms of discriminant validity, many researchers have started the important task to separate egalitarian approaches from other ideologies such as assimilation, e.g., Guimond et al., 2014; Hahn et al., 2015). This reasoning can be illustrated through the discussion of Study 2A on the merits of equating the manipulations on the dimensions considered therein: As foreshadowed above, one could take the stance that positive intercultural frames and/or demand effects, far from being undesirable confoundings, might be factors central to reducing prejudice in real-world settings: If valence and/or demand effects are identified as core psychological constructs in this way, then the next logical step would be to develop more effective framings of positive intergroup relationships and/or demand effects than the current categorization-centered frames, in which these constructs are only manipulated accidentally.

As for the prejudice reduction interventions aimed at reducing or enhancing categorization, there are doubtlessly several other ways to reassess their differential effectiveness in reducing prejudice.^4^ We ultimately concur with Wolsko et al.’s (2000, p. 649) insight that it is likely that “improved interethnic relations will depend both on the greater appreciation of group differences and on the treatment of one another as individual members of a common humanity.” Yet, we believe that the need for research on the right combination of these ingredients and on the ways to achieve that goal is still large.

## Supplemental Material

sj-docx-1-psp-10.1177_01461672251328704 – Supplemental material for Reconsidering Categorization-Related Inferences from Multicultural and Colorblind Prejudice Reduction Interventions Supplemental material, sj-docx-1-psp-10.1177_01461672251328704 for Reconsidering Categorization-Related Inferences from Multicultural and Colorblind Prejudice Reduction Interventions by Manuel Becker, Sarah Teige-Mocigemba, Jeffrey W. Sherman and Karl Christoph Klauer in Personality and Social Psychology Bulletin

## Notes

1. Note that a response sheet from the perspective manipulation, the self-report data, and the demographic data from one participant, each, were lost.

2. Most importantly, we refer to “Germany and Europe” instead of the United States, and to “the tenets of equality and nondiscrimination anchored in our constitution” instead of “our creed in the Declaration of Independence that ‘all men (and women) are created equal’.” As mentioned above, the German texts can be found at the online repository, together with the original text with marked changes.

3. Note that we also exchanged one word in the introduction of the tasks after participants had read the manipulation texts so that participants could understand all of the subsequent tasks even in the condition in which ethnic groups are not specifically named anymore. The introduction now reads: “The present text has been implemented in several scientific studies to encourage a certain perspective on different ethnic groups living together.”

4. It is also worth reiterating the argument considered in the Introduction that there may be other reasons besides effectiveness in reducing the majority group’s immediate prejudice to favor or abandon a perspective on interethnic relations.
