# Supplementary material for: Reconsidering Categorization-Related Inferences from Multicultural and Colorblind Prejudice Reduction Interventions
Source: Pers Soc Psychol Bull. 2025 Apr 16;52(7):1951–65. doi: 10.1177/01461672251328704 (PMC13216559; doi:10.1177/01461672251328704)
Supplement: sj-docx-1-psp-10.1177_01461672251328704 – Supplemental material for Reconsidering Categorization-Related Inferences from Multicultural and Colorblind Prejudice Reduction Interventions [file sj-docx-1-psp-10.1177_01461672251328704.docx]

**Reconsidering categorization-related inferences from multicultural and colorblind prejudice reduction interventions**

**Online Supplement for Study 1**

**Methods**

**Pilot Study**

*N*=22 participants (73% female, mean age = 23.8 years) were instructed to rate the behaviors on a scale from 1 (very typical for Turks) to 7 (very typical for Germans). As in the WSW procedure implemented in the following studies, in half of the statements, the positive version of a behavior was shown by means of random selection. A subsequent t-test showed no difference for the negative or positive versions of the statements with regard to their (stereo-)typicality (*M_neg_*= 4.05, *SD* = .54; *M_pos_*= 4.12, *SD* = .70; *t*<1, *p*=.39).

**Participant Reimbursement (Study 1B)**

Participants were paid 3 Eurocents for each time they correctly recognized that a behavior had not been presented in the discussion phase, and 12 Eurocents for each time they correctly assigned a behavior that had been presented in the discussion phase to the correct target. Participants did not know about the specific reimbursement rule, but did know that they would be paid out an extra 3 Euros unconditionally for taking part in the experiment.

**Evaluation Measures**

In Study 1A, a short self-report evaluation measure was implemented after the WSW recognition phase. All portraits (targets) from the WSW were presented again and had to be rated on four 7-point semantic differential scales. The endpoints of the scales were cold and warm, bad and good, unpleasant and pleasant, and dislikeable and likeable (German: “kalt“ and “warm“, “schlecht“ and “gut“, “unangenehm“ and “angenehm“, and “unsympathisch“ and “sympathisch“). Participants were presented with scales blocked per portrait in a fixed order. Prejudice scores were computed by subtracting the mean ratings of the Turkish portraits across scales from the ratings of German portraits. Accordingly, positive values indicate a preference for German over Turkish stimuli.

In Study 1B, a relatively standard version of the Evaluative Decision Task (Fazio, et al., 1995) was implemented between the WSW impression formation and recognition phases. In this task, participants underwent four blocks of trials: The first block consisted of 24 trials, in which participants were to get acquainted with the task. The following three blocks consisted of 48 test trials, each, in which a smaller version of each of the portrait stimuli shown in the WSW impression formation phase preceded two positive and two negative target words. The target words presented after the primes were sampled without replacement for each participant and block anew from a pool of 64 strongly polarized positive and 64 strongly polarized negative adjectives (see, e.g., Klauer et al., 2009). Each trial began with the presentation of a fixation cross for 250 ms, directly succeeded by the prime stimulus (i.e., the exemplar). With a stimulus-onset asynchrony of 200 ms, a target word replaced the prime stimulus and remained on the screen for 800 ms or until an evaluation response had been given by participants by clicking on either of two response buttons labeled as positive or negative. After an intertrial interval of 500 ms, the next trial was initiated. Before each test trial block, participants were presented with four warm-up trials, which, like the first block as a whole, do not factor into the measurement outcome. As described in the main body of the manuscript, participants were excluded from analysis if they had commited an unsual amount of errors in the Evaluative Decision Task (errors in more than 25% of trials). The subsequent analyses were based on log-transformed latencies of trials with correct responses, if latencies were not outliers in the participants’ latency distribution of correct responses, that is, if latencies were 1.5 times the interquartile range below the first, or 1.5 times the interquartile range above the third quantile in the corresponding distribution (cf. Tukey, 1977). Prejudice scores were computed by subtracting the mean latency of so-called congruent trials (where Turkish primes are succeeded by negative targets or where German primes are succeeded by positive targets) from incongruent trials (i.e., the remained of trials). Positive values of this analysis again indicate a preference for German over Turkish stimuli.

As discussed in the main body of the present manuscript, we believe that the validity of the operationalization of colorblind and multicultural perspective manipulations provided by Wolsko and colleagues (2000) is severely threatened, as they do not appear to produce the intended differential levels of social categorization, while producing unintended differences in the extent to which intergroup relations are portrayed as beneficial. Accordingly, we believe that the operationalizations’ effect on evaluation measures reported below is not a valid test for the effects of the underlying perspective on reducing prejudice. Thus, we present these results in this Appendix and refrain from interpreting then. As mentioned in the main body of the present manuscript as well, we collected these data as part of a different project.

**Who Said What? Paradigm**

A comprehensive description of the procedural details of the WSW is given in the main body of this manuscript. As described there, the target stimuli were selected from a stimulus pool provided by Singmann and colleagues (2013), who also conducted a comprehensive rating study of the stimuli. The stimuli for Study 1A were selected such that they were matched with regard to typicality of their respective social group (*M_Turks_* = 4.32, *SD_Turks_* = .53; *M_Germans_* = 4.28, *SD_Germans_* = .54; *t*[10] = .14, *p* = .89), valence of emotional expression (*M_Turks_* = 4.33, *SD_Turks_* = .65; *M_Germans_* = 4.34, *SD_Germans_* = .59; *t*[10] = 0.02, *p* = .98), and attractiveness (*M_Turks_* = 2.67, *SD_Turks_* = .27; *M_Germans_* = 2.80, *SD_Germans_* = .34; *t*[10] = 0.77, *p* = .46; see Teige-Mocigemba et al., 2017, which used exactly the same set of stimuli). For Study 1B, we exchanged one portrait typical of Turkish ethnicity, and one portrait typical of German ethnicity. In this selection, again, stimuli were matched with regard to typicality of their respective social group (*M_Turks_* = 4.28, *SD_Turks_* = .51;*M_Germans_* = 4.24, *SD_Germans_* = .52; *t*[10] = .12, *p* = .91), valence of emotional expression (*M_Turks_* = 4.57, *SD_Turks_* = .30; *M_Germans_* = 4.56, *SD_Germans_* = .29; *t*[10] = 0.08, *p* = .94), and attractiveness (*M_Turks_* = 2.72, *SD_Turks_* = .20; *M_Germans_* = 2.86, *SD_Germans_* = .23; *t*[10] = 1.17, *p* = .27).

As described in the main body of this manuscript, the participants‘ recognition performance is analyzed by means of a mathematical model proposed by Klauer & Wegener (1998). In the following, details of this analysis will be reiterated, in part reproducing descriptions from Klauer and colleagues (2014). For the sake of simplicity, the descriptions presented here refer to the baseline model. As explained below and in the main body of the manuscript, the hypothesis tests are based on a slightly more complex model where person memory varies as a function of stimulus ethnicity.

Figure 1 shows a graphical depiction of part of the model. The figure shows the processing tree for a negative behavior shown by a Turkish target. It depicts the processing events leading to the four response categories that can arise for this kind of behavior made by Turkish targets. Analogous processing trees account for the assignments of positive behaviors and of behaviors of either kind from German targets. The response categories are displayed as rectangular boxes on the right hand side of the graph. The responses are classified as either a correct assignment when the correct target is chosen, within-category errors, between-category errors, and false rejections (the old behavior is falsely judged new).

An item will be recognized as old with probability *I* for item memory or not with probability 1 − *I*. In the event of item memory, its target will be remembered with probability *c* for person memory, in which case the statement is assigned to the correct person. In case the correct person is not remembered (with probability 1 − *c*), there might still be category memory for the target’s ethnic category with probability *d* or not with probability 1 − *d*. In the event of category memory, the correct target can still be guessed with a fixed probability of 1/6 since there are six targets in each category in the present studies. If the correct target is not guessed with probability 5/6, a within-category error occurs. If category membership is not retrieved with probability 1 − *d*, on the other hand, participants are disposed to assign a negative behavior to a Turkish target rather than a German target with probability *a_1_*. A value of *a_1_* = .5 corresponds to the absence of a systematic bias to prefer one or the other category in guessing a target; a value larger than .5 indicates a preference for assigning negative behaviors to a Turkish target; a value smaller than .5 indicates a preference for Geman targets. If a target from the correct category is guessed, it will be the correct target with probability 1/6, whereas a within-category error occurs otherwise. If a target from the false category is guessed, a between-category error occurs.

**Figure 1**

*Multinomial Model for the WSW task*
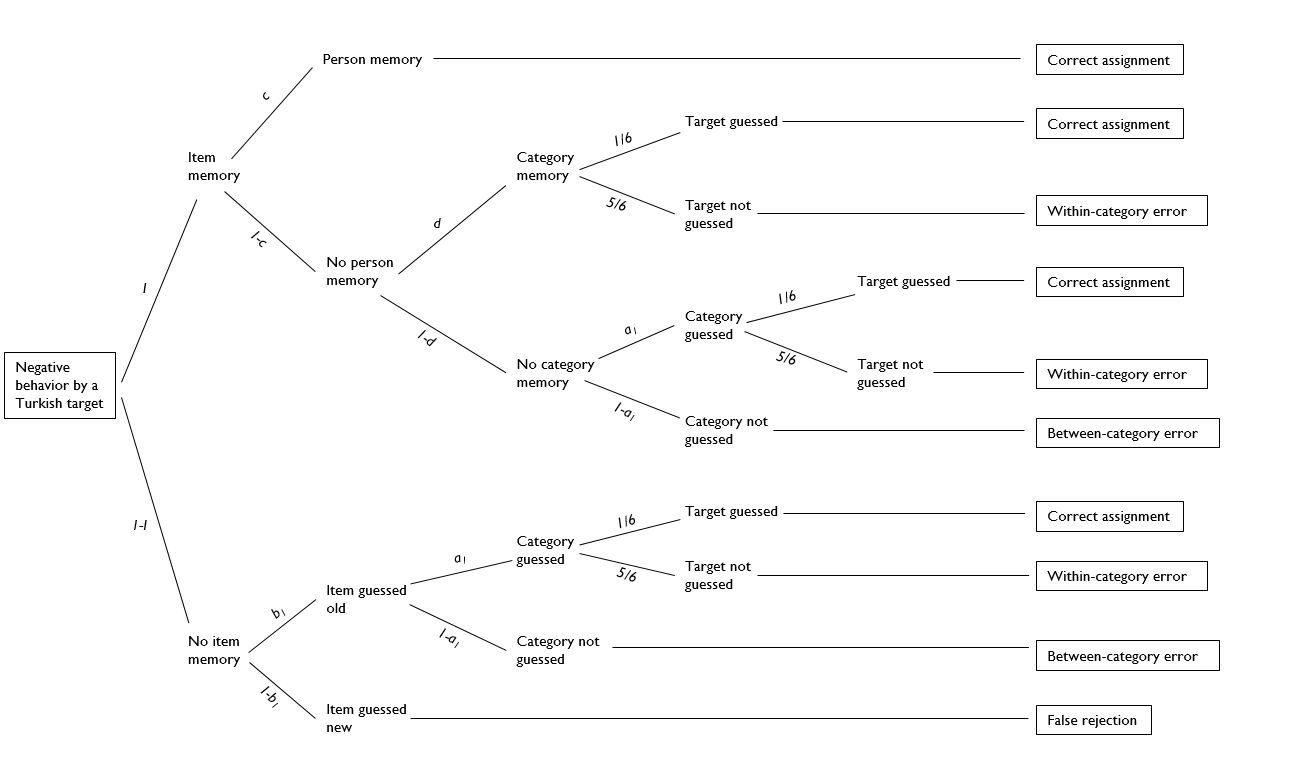


*Note*. The rectangles indicate manifest states (i.e., item type and responses), all other variables indicate latent states.

Traversing the tree in this way accounts for all the different processing paths by which one of the different responses can occur. The processing tree for distracters is much simpler, as there are no category-memory and person-memory processes involved in assigning items that were not presented. Responses are classified by behavior valence, source (Turkish target, German target, distracter), and the four response categories shown on the right of Figure 1, for a total of 22 response categories (note that there are only three response categories for distractor trees given that a correct assignment to a target cannot occur for these).

The model specifies the probabilities with which the different response categories are expected to occur given numerical values for the different model parameters. For example, a correct assignment of a negative behavior by a Turkish target is expected to occur with probability

*P*(correct assignment|Negative behavior by a Turkish target) =

*Ic* +

*I*(1 − *c*)*d* $\frac{1}{6}$ +

*I*(1 − *c*)(1 − *d*)*a_1_* $\frac{1}{6}$ +

(1 − *I*)*ba_1_* $\frac{1}{6}$ ,

each row of the equation corresponding to one of the four paths of the tree in Figure 1 that lead to a correct assignment.

The observed relative response frequencies estimate these response probabilities. On the basis of the estimated response probabilities and the model equations, model parameters can be estimated. Parameter values are chosen so as to reproduce the estimated probabilities as closely as possible. This is often done via maximum-likelihood estimation aggregating across items (i.e., statements) and participants (Batchelder & Riefer, 1999).

**Multilevel Extension of the Model**

As recently explained by Judd, Westfall, and Kenny (2012), substantial biases are, however, inherent in analyses that ignore one or the other of these random factors (i.e., items or participants), and the standard approach ignores both. We therefore used a Bayesian approach proposed by Klauer (2010) and Matzke and colleagues (2015) to fit a multilevel extension of the model that treats participants and items as random factors for each model parameter.

For a multilevel extension of the model, random effects for participants and items are considered for each parameter to account for systematic differences between participants and items. For example, considering item memory, participants may differ in the extent to which they memorize the items, and items may differ in memorability. In the multi-level extension, the item-memory parameter *I* is given as *I_ij_*, where *i* indexes participants, and *j* indexes items with

*I_ij_ = G(μ_I_ + α_i_ + β_j_),* (1)

where *μ_I_* is the population mean for item memory, α*_i_* is the *i*th participant effect, and *β_j_* is the *j*th item effect on item memory. The function *G* establishes a link between these effects and item memory. Such a link is necessary because item memory is a probability, constrained to range between 0 and 1, while the additive effects are real valued (Pratte & Rouder, 2012). Following Klauer (2010) and Matzke et al. (2015), we used a probit link, that is the inverse cumulative distribution function of a standard normal.

Remember that the baseline model sets equal parameters *I*, *c*, *d*, and *b* across statement type, and item effects for items were therefore also set equal across statement type (i.e., entered per statement pair) for these parameters. Failing to do so would have re-introduced differences in these parameters as a function of statement type via the backdoor of item effects.

Further constraints are gained by imposing hierarchical structures on participant and item effects for all model parameters. Participant and item effects are assumed to follow multivariate normal distributions with zero mean and a variance-covariance matrix that is a free parameter to be estimated from the data. There is one such matrix for the participant effects, and a separate one for item effects. These structures pull large effects toward zero, mitigating the influence of outliers. The variance-covariance matrices allow one to account for and estimate correlations between parameters across items and participants.

Estimation of the multilevel model can in principle be done via maximum-likelihood estimation, but the computational complexity of maximum-likelihood estimation is prohibitive for multilevel models (Klauer, 2010). We therefore used a Bayesian approach that is computationally tractable and converges with the maximum-likelihood estimates for large data samples. The Bayesian approach requires one to specify so-called hyperprior distributions for the condition effects and variance-covariance matrices. Here, we worked with the uninformative hyperpriors proposed by Matzke et al. (2015) and Klauer (2010).

**Modeling decisions**

As described above, the model allows one to assess social categorization (in the form of category memory) disentangled from other mental processes (see Table 3). In applying the model, a number of decisions have to be made, which also influence the number of parameters that are estimated in the model. As in Klauer and colleagues (2003, 2014), only category guessing parameters were allowed to vary as a function of behavior valence (see above). In addition, each memory parameter can be estimated as a function of the ethnicity of the stimulus (i.e., of the person) that had originally shown the behavior. For Study 1A, we estimated six models: the simplest model, in which all memory parameters (I, c, d; see Table 3) are set equal across ethnicities; two models in which either person memory or category memory were allowed to vary as a function of stimulus ethnicity; and two models in which item memory was allowed to vary as a function of ethnicity. The difference between these last two models is whether distractor behaviors were jointly estimated with behaviors from Turks or behaviors from Germans. In a final model, category memory varied not only by stimulus ethnicity, but also by the valence of the behavior shown by the stimulus. We then compared model fit for all six models with the Watanabe-Akaike Information Criterion (WAIC, Watanabe, 2010; cf. Vehtari et al., 2017), a Bayesian model evaluation criterion replacing the popular Deviance Information Criterion (DIC, Spiegelhalter et al., 2002). The model in which person memory parameters were allowed to vary as a function of stimulus ethnicity provided the best relative fit for the data (*WAIC*=21412.9). We therefore chose this model for the following analyses of the individual study parts 1A&B, and of a joint analysis of their data.

**Model fit and parameter estimation**

The Bayesian hierarchical method described above provides three model fit estimates (i.e., *T_1_*, *T_2a_*, and *T_2b_*). *T_1_* corresponds to the goodness-of-fit statistic *G^2^* used in the conventional multinomial modeling approach introduced by Batchelder and Riefer (1999). It estimates how well the pattern of the observed assignment frequencies within each condition and aggregated over participants and items is accounted for by the model. The model also provides two summary statistics for how well the model accounts for variances and correlations of the observed assignment frequencies within each condition when participants (*T_2a_*) or items (*T_2b_*) are taken as units. *T_2b_* suggested significant deviations of the applied model from the data, so we computed individual model fit statistics for every participant, leading to the exclusion of four participants, as mentioned above. After their exclusion, model checks for the individual studies and the joint analysis were satisfactory, and no further participant had to be excluded from analyses, see Table 1.

**Table 1**

*Model checks for Studies 1A and 1B*

| Study | Parameter | *Observed* | *predicted* | *P* |
| --- | --- | --- | --- | --- |
|  | *T_1_* | 52.2 | 58.4 | .69 |
| 1A | *T_2a_* | 2522 | 2478 | .42 |
|  | *T_2b_* | 1186 | 1172 | .43 |
|  | *T_1_* | 75.0 | 58.4 | .10 |
| 1B | *T_2a_* | 2963 | 2688 | .26 |
|  | *T_2b_* | 1255 | 1179 | .24 |
|  | *T_1_* | 79.1 | 58.5 | .05 |
| Joint | *T_2a_* | 2827 | 2636 | .28 |
|  | *T_2b_* | 2765 | 2420 | .05 |

The hierarchical Bayesian approach yields Highest Density Intervals (HDIs) for all parameter estimates, similar in interpretation to standard confidence intervals. More importantly for the present purposes, it also allows for hypothesis tests for equality between parameters across the different experimental conditions. These are based on the samples from the posterior distribution of the parameters given the data that the Bayesian analysis provides. These samples allow one to estimate how likely the parameter values in one condition are to be larger than the values in another condition; we present these posterior comparisons as a *p* value. For the present purposes, the key comparison tested for equality of the category memory parameter d in the multicultural and colorblind conditions. We also compared the three conditions in which, in our reading, attention was directed towards social categories with the condition in which attention was directed towards individual behavior.

**Outcomes of the analyses**

The hierarchical Bayesian approach yields Highest Density Intervals (HDIs) for all parameter estimates, similar in interpretation to standard confidence intervals. More importantly for the present purposes, it also allows for hypothesis tests for equality between parameters across the different experimental conditions. These are based on the samples from the posterior distribution of the parameters given the data that the Bayesian analysis provides. These samples allow one to estimate how likely the parameter values in one condition are to be larger than the values in another condition; we present these posterior comparisons as a *p* value. For the present purposes, the key comparison tested for equality of the category memory parameter d in the multicultural and colorblind conditions. We also compared the three conditions in which, in our reading, attention was directed towards social categories with the condition in which attention was directed towards individual behavior.

**Computing Bayes factors**

For the model defining *H_1_* of the Bayes-factor based model comparison, effects of the perspective manipulations are admitted for parameters *a_1_* and *a_2_* for guessing category membership given a positive or negative behavior, respectively, for parameter *c* for person memory, and for category memory *d*. To examine the effects of the manipulations on the category memory parameter, they were contrast-coded by means of three contrasts. For the two theoretically interesting contrasts (multicultural vs. colorblind perspective manipulation; multicultural and colorblind perspective manipulations and control group “existence of different groups” versus control group “individual behavior”), Bayes factors were computed. To this end, contrasts were effect-coded by means of an effect size parameter δ such that $\frac{1}{2}$ δ increases the parameter values with positive contrast weights and decreases parameter values for condition with negative contrast weights also by $\frac{1}{2}$ δ. A prior for this effect-size parameter specifies the expected effect sizes. Following Gronau and colleagues (2019), it is chosen as a normal distribution with mean 0 and standard deviation 0.5244005. As explained by Gronau et al. (2019), this corresponds to the expectation that the effect of the manipulations would not exceed a difference of 0.4 on the probability scale if it were centered on 0.5 (i.e., the values in the contrasted conditions would then be 0.3 and 0.7). All tests were conducted as one-tailed hypothesis tests, and therefore, the normal distribution is truncated from below at zero. The Bayes factor is computed using the Savage-Dickey method (Wagenmakers et al., 2010).

**Results**

**Evaluation Measures**

***Self-report Measure***

As described above, positive values in the prejudice score reported in Table 2 indicate a preference for German over Turkish stimuli. As can be seen, all conditions report descriptively negative values. An analysis of variance where the measurement outcome is analyzed as a function of the between-participants factor condition reveals no significant main effect, *F*(3,156) = .64, *p* = .59.

**Table 2**

*Condition-wise self-report prejudice scores in Study 1A*

| Condition | Mean Evaluative Preference for Germans | *SD* | *d* |
| --- | --- | --- | --- |
| Multicultural Perspective Manipulation | -0.32 | 0.73 | -.44 |
| Colorblind Perspective Manipulation | -0.41 | 0.73 | -.57 |
| Control Group “Existence of different groups“ | -0.31 | 0.79 | -.39 |
| Control Group “Individual Behavior“ | -0.19 | 0.64 | -.30 |

***Evaluative Decision Task***

As described above, positive values in the prejudice score reported in Table 3 indicate a preference for German over Turkish stimuli. As can be seen, all conditions report descriptively positive values. An analysis of variance where the measurement outcome is analyzed as a function of the between-participants factor condition reveals no significant main effect, *F*(3,153) = .9, *p* = .45.

**Table 3**

*Condition-wise Evaluative Decision Task prejudice scores in Study 1B*

| Condition | Mean Evaluative Preference for Germans | *SD* | *d* |
| --- | --- | --- | --- |
| Multicultural Perspective Manipulation | 0.4 | 18 | .03 |
| Colorblind Perspective Manipulation | 1.0 | 14 | .09 |
| Control Group “Existence of different groups“ | 5.9 | 17 | .38 |
| Control Group “Individual Behavior“ | 2.3 | 20 | .12 |

*Notes*. The mean evaluative preference (congruency effect) and its standard deviation are reported in milliseconds. The Cohen’s d statistic refers to log-transformed response times.

***Who Said What? paradigm***

**Model Fit**. As described in the main body of the manuscript, the Watanabe-Akaike Information Criterion (WAIC) is currently described as the most adequate information criterion for assessing model fit of Bayesian models. When applied to the data of Experiment 1A, the model where person memory varies as a function of stimulus ethnicity emerges as the model with the best relative fit to the data, followed by the model where category memory varies as a function of stimulus ethnicity, cf. Table 4.

**Table 4**

*Model fit estimates for Study 1A*

| Model | *WAIC* | *SE* |
| --- | --- | --- |
| Simple model | 21442.3 | 197.5 |
| Person memory varies as function of stimulus ethnicity (*c_t_*, *c_g_*) | 21412.9 | 197.7 |
| Category memory varies as function of stimulus ethnicity (*d_t_*, *d_g_*) | 21435.2 | 197.4 |
| Item memory varies as function of stimulus ethnicity, distractors and behaviors from Turks modeled jointly (*I_tn_*, *I_g_*) | 21468.8 | 197.6 |
| Item memory varies as function of stimulus ethnicity, distractors and behaviors from Germans modeled jointly (*I_t_*, *I_gn_*) | 21466.0 | 197.5 |
| Category memory varies as function of stimulus ethnicity and behavior valence (*d_t+_*, *d_g+_*_,_ *d_t-_*, *d_g-_*) | 21462.3 | 197.4 |

**Model parameters.** Table 5 recovers the parameters from both studies and the joint analysis, along with 95% highest density intervals, for said model where person memory varies as a function of stimulus ethnicity. The parameters are reported for each condition individually.

**Table 5**

*Parameter estimates for both studies and the joint analysis*

| Parameter | Analysis Study 1  (and 95% HDI intervals) | | | Analysis Study 2  (and 95% HDI intervals) | | | Joint Analysis  (and 95% HDI intervals) | | |
| --- | --- | --- | --- | --- | --- | --- | --- | --- | --- |
| Multicultural Condition | | | | | | | | | |
| *a1* | (.42) | **.49** | (.56) | (.39) | **.46** | (.54) | (.43) | **.48** | (.53) |
| *a2* | (.62) | **.69** | (.77) | (.55) | **.62** | (.70) | (.60) | **.65** | (.71) |
| *B* | (.03) | **.06** | (.11) | (.07) | **.12** | (.18) | (.06) | **.09** | (.13) |
| *c_d_* | (.30) | **.38** | (.47) | (.38) | **.45** | (.52) | (.35) | **.41** | (.47) |
| *c_t_* | (.22) | **.29** | (.36) | (.24) | **.31** | (.39) | (.25) | **.30** | (.35) |
| *D* | (.35) | **.43** | (.52) | (.30) | **.39** | (.48) | (.35) | **.41** | (.47) |
| *I* | (.75) | **.80** | (.85) | (.74) | **.80** | (.85) | (.76) | **.80** | (.84) |
| Colorblind Condition | | | | | | | | | |
| *a1* | (.39) | **.46** | (.53) | (.37) | **.44** | (.52) | (.40) | **.45** | (.51) |
| *a2* | (.65) | **.73** | (.79) | (.61) | **.69** | (.76) | (.65) | **.70** | (.75) |
| *B* | (.03) | **.05** | (.09) | (.05) | **.09** | (.15) | (.42) | **.07** | (.10) |
| *c_d_* | (.28) | **.36** | (.45) | (.32) | **.39** | (.47) | (.31) | **.37** | (.43) |
| *c_t_* | (.20) | **.27** | (.34) | (.24) | **.32** | (.40) | (.24) | **.29** | (.34) |
| *D* | (.32) | **.40** | (.49) | (.31) | **.40** | (.49) | (.35) | **.41** | (.47) |
| *I* | (.72) | **.78** | (.83) | (.74) | **.80** | (.85) | (.74) | **.79** | (.83) |
| Control Group Groups | | | | | | | | | |
| *a1* | (.43) | **.50** | (.58) | (.35) | **.43** | (.49) | (.41) | **.46** | (.52) |
| *a2* | (.52) | **.60** | (.68) | (.55) | **.63** | (.70) | (.55) | **.61** | (.67) |
| *B* | (.04) | **.08** | (.13) | (.49) | **.09** | (.15) | (.05) | **.08** | (.12) |
| *c_d_* | (.28) | **.37** | (.45) | (.39) | **.47** | (.55) | (.35) | **.42** | (.47) |
| *c_t_* | (.21) | **.28** | (.35) | (.19) | **.26** | (.33) | (.22) | **27** | (.32) |

| Parameter | Analysis Study 1  (and 95% HDI intervals) | | | Analysis Study 2  (and 95% HDI intervals) | | | Joint Analysis  (and 95% HDI intervals) | | |
| --- | --- | --- | --- | --- | --- | --- | --- | --- | --- |
| Control Group Groups | | | | | | | | | |
| *D* | (.38) | **.47** | (.57) | (.30) | **.39** | (.48) | (.37) | **.43** | (.49) |
| *I* | (.72) | **.78** | (.84) | (.70) | **.76** | (.82) | (.72) | **.77** | (.82) |
| Control Group Individual | | | | | | | | | |
| *a1* | (.32) | **.39** | (.45) | (.37) | **.44** | (.51) | (.37) | **.42** | (.47) |
| *a2* | (.56) | **.63** | (.70) | (.54) | **.62** | (.69) | (.57) | **.62** | (.68) |
| *B* | (.03) | **.06** | (.10) | (.08) | **.14** | (.21) | (.06) | **.10** | (.14) |
| *c_d_* | (.31) | **.39** | (.48) | (.38) | **.45** | (.53) | (.36) | **.43** | (.49) |
| *c_t_* | (.23) | **.30** | (.38) | (.22) | **.29** | (.37) | (.25) | **.30** | (.35) |
| *D* | (.24) | **.33** | (.42) | (.25) | **.35** | (.44) | (.28) | **.34** | (.41) |
| *I* | (.70) | **.76** | (.82) | (.66) | **.72** | (.79) | (.69) | **.75** | (.79) |

*Notes*. *c_d_*: person memory for German stimuli, *c_t_*: person memory for Turkish stimuli

References

Batchelder, W. H., & Riefer, D. M. (1999). Theoretical and empirical review of multinomial

processing tree modeling. *Psychonomic Bulletin & Review, 6*, 57-86. https://doi.org/10.3758/BF03210812

Fazio, R. H., Jackson, J. R., Dunton, B. C., & Williams, C. J. (1995). Variability in automatic

activation as an unobtrusive measure of racial attitudes. *Journal of Personality and Social Psychology, 69*, 1013-1027. https://doi.org/10.1037/0022-3514.69.6.1013

Gronau, Q. F., Wagenmakers, E.-J., Heck, D. W., & Matzke, D. (2019). A simple method for

comparing complex models: Bayesian model comparison for hierarchical multinomial processing tree models using Warp-III bridge sampling. *Psychometrika, 84*, 261-284. https://doi.org/10.1007/s11336-018-9648-3

Judd, C., Westfall, J., & Kenny, D. A. (2012). Treating stimuli as a random factor in social

psychology: A new and comprehensive solution to a pervasive but largely ignored problem. *Journal of Personality & Social Psychology, 10*, 54-69. https://doi.org/10.1037/a0028347

Klauer, K. C. (2010). Hierarchical multinomial processing tree models: A latent-trait

approach. *Psychometrika, 75*, 70-98. https://doi.org/10.1007/s11336-009-9141-0

Klauer, K. C., Ehrenberg, K., & Wegener, I. (2003). Crossed categorization and stereotyping:

Structural analyses, effect patterns, and dissociative effects of context relevance. *Journal of Experimental Social Psychology, 39*, 332-354. <https://doi.org/10.1016/S0022-1031(03)00017-9>

Klauer, K. C., Hölzenbein, F., Calanchini, J., & Sherman, J. W. (2014). How malleable is

categorization by race? Evidence for competitive category use in social categorization. *Journal of Personality and Social Psychology, 107*, 21–40. https://doi.org/10.1037/a0036609

Klauer, K. C., Teige-Mocigemba, S. & Spruyt, A. (2009). Contrast effects in spontaneous

evaluations: A psychophysical account. *Journal of Personality and Social Psychology, 96*, 265-287. https://doi.org/0.1037/a0013248

Klauer, K. C., & Wegener, I. (1998). Unraveling social categorization in the “Who said

what?” paradigm. *Journal of Personality and Social Psychology, 75,* 1155-1178. https://doi.org/10.1037/0022-3514.75.5.1155

Matzke, D., Dolan, C. V., Batchelder, W. H., & Wagenmakers, E.-J. (2015). Bayesian

estimation of multinomial processing tree models with heterogeneity in participants and items. *Psychometrika, 80*, 1–31. https://doi.org/10.1007/s11336-013-9374-9

Pratte, M. S., & Rouder, J. N. (2012). Assessing the dissociability of recollection and

familiarity in recognition memory. *Journal of Experimental Psychology: Learning, Memory, and Cognition*, 38, 1591-1607. https://doi.org/10.1037/a0028144

Singmann, H., Kellen, D., & Klauer, K. C. (2013). Investigating the other-race effect of

Germans toward Turks and Arabs using multinomial processing tree models. In M. Knauff, M. Pauen, N. Sebanz, & I. Wachsmuth (Eds.), *Proceedings of the 35th Annual Conference of the Cognitive Science Society* (p. 1330-1335). Austin, TX: Cognitive Science Society.

Spiegelhalter, D. J., Best, N. G., Carlin, B. P. & Van der Linde, A. (2002). Bayesian Measures

of Model Complexity and Fit (with Discussion). *Journal of the Royal Statistical Society, Series B, 64*, 583-616. <https://doi.org/10.1111/1467-9868.00353>

Teige-Mocigemba, S., Becker, M., Sherman, J. W., Reichardt, R., & Klauer, K. C. (2017).

The Affect Misattribution Procedure: In search of prejudice effects. *Experimental Psychology, 64*, 215-230. <https://doi.org/10.1027/1618-3169/a000364>

Tukey, J. W. (1977). *Exploratory data analysis*. Reading, MA: Addison-Weasley.

Vehtari, A., Gelman, A., & Gabry, J. (2017). Practical Bayesian model evaluation using

leave-one-out cross-validation and WAIC. *Statistics and Computing, 27*, 1413–1432. <https://doi.org/10.1007/s11222-016-9696-4>

Wagenmakers, E.-J., Lodewyckx, T., Kuriyal, H., and Grasman, R. (2010). Bayesian

hypothesis testing for psychologists: A tutorial on the Savage-Dickey method. C*ognitive Psychology, 60*, 158-189. https://doi.org/10.1016/j.cogpsych.2009.12.001

Watanabe, S. (2010). Asymptotic equivalence of Bayes cross validation and widely applicable

information criterion in singular learning theory. *Journal of Machine Learning Research, 11*, 3571–3594.

Wolsko, C., Park, B., Judd, C. M., & Wittenbrink, B. (2000). Framing interethnic ideology:

Effects of multicultural and color-blind perspectives on judgements of groups and individuals. *Journal of Personality and Social Psychology, 78*, 635-654. https://doi.org/10.1037/0022-3514.78.4.635
